# Band Gap and Polarization Tuning of Ion-Doped *X*NbO_3_ (*X* = Li, K, Na, Ag) for Photovoltaic and Energy Storage Applications

**DOI:** 10.3390/molecules29051011

**Published:** 2024-02-26

**Authors:** Iliana N. Apostolova, Angel T. Apostolov, Julia M. Wesselinowa

**Affiliations:** 1 Universityof Forestry, 10, Kl. Ohridsky Blvd., 1756 Sofia, Bulgaria; inaapos@abv.bg; 2 University of Architecture, Civil Engineering and Geodesy, 1, Hristo Smirnenski Blvd., 1046 Sofia, Bulgaria; angelapos@abv.bg; 3 Department of Physics, Sofia University “St. Kliment Ohridski”, Blvd. J. Bouchier 5, 1164 Sofia, Bulgaria

**Keywords:** *X*NbO_3_, ion doping, band gap energy, polarization, microscopic model

## Abstract

Using a microscopic model and Green’s function theory, we have calculated the band gap energy and the polarization of LiNbO_3_, KNbO_3_, AgNbO_3_, and NaNbO_3_. The effects by substitution of different ions at A or/and B sites for doping concentration *x* = 0–0.1 are studied. The observed different tuning of these properties is discussed for the possibility of photovoltaic and energy storage applications of these compounds. They should have a large polarization and narrow band gap. It is shown that the band gap of all substances decreases or increases with increasing Fe or Zn dopant at the Nb site, respectively. But the substitution, for example, of Ba at the A site, leads to different behaviors of these materials. The polarization increases by Ba doping at the A site and decreases by Fe doping at the Nb site. For example, by Ba/Fe, Ba/Ni co-doping (Ba at the A site and Fe, Ni at the B site) we observe both an enhanced polarization and reduced band gap.

## 1. Introduction

In the realm of advanced materials for photovoltaic applications, the interplay between ferroelectric polarization and band gap characteristics plays a pivotal role in enhancing the bulk photovoltaic response. This phenomenon, which involves the generation of a photocurrent in materials possessing a polar non-centrosymmetric structure like ferroelectric perovskite oxides, has garnered significant attention in recent research endeavors. Among the diverse array of perovskite oxides under investigation, two lead-free variants, namely LiNbO_3_ (LNO) and KNbO_3_ (KNO), have emerged as particularly promising candidates. These materials exhibit intriguing properties that make them highly attractive for various optical applications, with a specific focus on bulk photovoltaic functionalities. One of the key factors contributing to the appeal of LNO and KNO lies in their tunable characteristics. Through techniques such as strain engineering, controlled doping, or the application of an external electric field, both the band gap and ferroelectric polarization of these materials can be effectively tailored [1,2]. This tunability opens up avenues for fine-tuning the material properties to meet specific application requirements, thereby expanding their potential utility in photovoltaic technologies. The ability to manipulate the band gap allows for the optimization of the absorption properties of these materials, thereby enhancing their efficiency in converting incident light into electrical energy. Simultaneously, ferroelectric polarization plays a crucial role in facilitating charge separation and transport within the material, which are essential processes for efficient photovoltaic performance. Given these advantageous properties, ongoing research efforts are focused on further elucidating the underlying mechanisms governing the photovoltaic response of LNO and KNO-based ferroelectrics. Additionally, exploration into novel fabrication techniques and device architectures aims to harness the full potential of these materials for practical photovoltaic applications. The unique combination of a large ferroelectric polarization and a tunable band gap in materials such as LNO and KNO holds immense promise for advancing the field of bulk photovoltaics.

The origin of this tuning is not fully explained. Moreover, the results for the band gap energy $E_{g}$ and the polarization *P* of ion-doped LNO or KNO are still controversial. Therefore, we will try to clarify which doping ions reduce $E_{g}$ and which enhance *P*. Unfortunately, there is not much experimental data on the effect of ion doping on the band gap and the polarization. LNO has been widely used as a pyroelectric material due to its spontaneous electric polarization, and as a nonlinear optical material [3]. KNO is one of the candidate materials for lead-free piezoelectric applications because of its large piezoelectricity and high Curie temperature [4].

It must be noted that K^+^, Li^+^, and Nb^5+^ are paramagnetic ions. Therefore, pure bulk KNO or LNO are nonmagnetic, they are only ferroelectric. However, it is observed that KNO and LNO nanoparticles show a ferromagnetic behavior [5,6]. The magnetization increases with decreasing nanoparticle size. Moreover, transition metal and rare earth ion-doped bulk KNO and LNO also exhibit ferromagnetism, so they are promising candidates for room temperature multiferroic applications [7,8].

Pure KNO shows semiconducting properties with a bandwidth of about 3.2–3.3 eV [9]. Transition metal ion doping at the Nb site can lead to a decreasing band gap energy [10,11,12]. Electronic and optical properties of LNO under tensile and compressive strain for optoelectronic applications are investigated from Density Functional Theory (DFT) computations by Raturi et al. [13]. First-principles calculations to investigate the electronic structures and band gap energies of KNO with 3d transition metal substitution at the Nb site were conducted by Liang and Shao [14]. The authors show that perovskite oxides are potential key materials for photovoltaic and transparent photonic applications. Using first-principles calculations, the electronic and optical properties of Ag and Au-doped LNO are investigated. For Au- and Ag-doped LNO, Zainuddin et al. [15] calculated a smaller band gap value compared with the pure one. Using the DFT, Jameel et al. [16] showed that Sr-doped KNO is an appropriate material for perovskite solar cell applications. The effect of defects on the spontaneous polarization in pure and doped LNO from first principle calculations were studied by Wang et al. [17]. The band gap of pure LNO is about 4–4.2 eV [18,19]. It can be modified by different ion doping [20,21,22,23]. Let us emphasize that both ions, Nb and K(Li), could also be substituted with different ions [24]. It should be noted that there is not much experimental data for the band gap values in ion-doped LNO and KNO. Most works are theoretical ones using the DFT, for example, Liang and Shao [14] reported $E_{g}$ values for pure, Fe-dope, and Zn-doped KNO: 2.74, 1.25, 3.726 eV, respectively. Huang et al. [25] observed, for pure and Fe-doped LNO, $E_{g}$ = 3.527 and 2.25 eV, respectively.

AgNbO_3_ (ANO) and NaNbO_3_ (NNO) are two noteworthy compounds characterized by their isostructural antiferroelectric behavior. Belonging to the perovskite family, these materials exhibit a distinctive orthorhombic symmetry, specifically within the Pbcm space group, when observed at ambient temperatures [26]. This orthorhombic structure is a result of the arrangement of atoms within the crystal lattice, where the oxygen octahedra formed by the NbO6 units are distorted due to the presence of Ag or Na cations. These distortions play a crucial role in determining the unique electronic and dielectric properties exhibited by ANO and NNO. Antiferroelectricity, a phenomenon observed in these materials, refers to the presence of alternating electric dipoles in adjacent unit cells that cancel each other out macroscopically. This behavior arises from the displacement of ions within the crystal lattice, leading to the formation of electrically polarized domains. ANO is a lead-free piezoelectric material with potential applications in electronic technology and catalysis [27]. The band gap of ANO is about 2.8 eV while that of NNO is about 4 eV. This is the same problem, the band gap energies are too large for application in solar photovoltaics. They could be reduced by different ion dopings [13]. Li et al. [24], using DFT calculations, have shown that in Co-doped NNO, the photoabsorption edge was shifted to the visible light region. The dopant properties of ANO were investigated by Kuganathan et al. [28]. La-substituted ANO for photocatalytic applications was studied by Khor et al. [29]. Eg decreases also by Ba-doped ANO [30] and Ca-doped NNO [31], whereas it increases by Mg and Ca doping of NNO [32]. The electronic structure of ANO and NNO was studied by X-ray photoelectron spectroscopy by Kruczek et al. [26]. This ability to tailor the cation composition opens avenues for enhancing the materials’ charge transport mechanisms, polarization characteristics, and electrochemical performance, crucial for applications ranging from solar cells to electrochemical capacitors. Additionally, exploring various combinations of A and B site cations enables the creation of multifunctional materials with synergistic properties, further broadening their potential applications in energy conversion and storage [33,34]. Understanding the intricate relationships between cation modifications and material properties facilitates the development of novel strategies for improving the efficiency, stability, and sustainability of energy storage technologies.

Because it is difficult to find a doping ion that reduces the band gap and increases the polarization, in the last few years, there have been investigations with two or three co-doping ions at different sites. For example, Zhang et al. [3] reported the modulation of ferroelectric and optical properties of La/Co-doped KNO, where Co doping is responsible for the obvious reduction of the band gap, whereas the La doping can enhance the polarization. One can conclude that co-doping on different sites, and its potential for the simultaneous tuning of band gap and polarization could open up exciting avenues for future exploration and optimization.

Finally, it could be mentioned that in order to understand the properties of ferroelectric materials and make a comparative analysis with other theoretical works, it could be of interest to study the intricate interplay of distortions and vacancies in these materials [35] or to investigate their pyroelectric properties with potential applications in energy harvesting and microelectronic devices [36].

The aim of the present work is to use the s-d model and Green’s function theory to investigate the band gap and polarization tuning in *X*NbO_3_, (*X* = K, Li, Ag, Na) by different ion doping, which is appropriate for photovoltaic and energy storage applications. It should be noted, that most theoretical works are made using the DFT, which is a highly potent tool for exploring many-body problems. However, DFT predominantly focuses on ground state properties at zero temperature. In contrast, our approach encompasses the entire temperature range, providing a finite temperature analysis that spans the complete excitation spectrum. Notably, our methodology enables the examination of the total phase diagram, which is constructed based on the various excitation energies exhibited by the system. One drawback of our approach is its initial consideration of collective properties, as opposed to focusing on individual electrons as in DFT. We work with effective spins of the underlying quasi-particles from the outset. While DFT theoretically allows for the calculation of all system parameters, we are compelled to employ additional models to determine these parameters. We firmly believe that both DFT and the Green’s function method, which we employ, are well-suited and, to some extent, offer alternative perspectives for describing many-body systems.

## 2. Results and Discussion

### 2.1. Model

**Figure 1:**
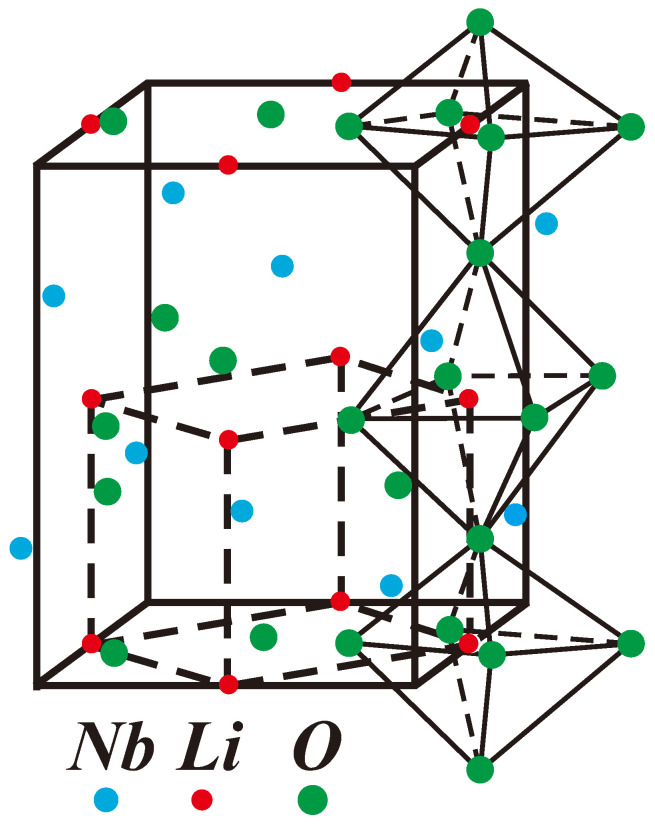
(Color online) Structure of LiNbO_3_ (LNO).

LNO is a typical representative of displacive-type ferroelectrics, belonging to the perovskite family with a structural formula ABO_3_, where A is an alkali or rare earth element, and B is a transition metal. In the paraelectric phase, the perovskite structure has a cubic centrosymmetric arrangement. A ions occupy the corners of the cube, B ions are located at the center of the body diagonal, and oxygen atoms are positioned at the intersection point of the diagonals on the cube’s faces (see Figure 1). Polarization is observed when the B atom is displaced from the center of the cube along one of its symmetry axes. In these ferroelectrics, dipole moments are created at the point of the phase transition. The ferroelectric states in ABO_3_ arise due to long-range dipole forces that destabilize the nonpolar phase. The elementary cell of LNO contains four Li^3+^ and Nb^3+^ cations along with twelve O^2−^ anions. It consists of interconnected LiO_6_ octahedra, where the positions of Nb cations are localized at the center of the twelve closest oxygen anions. The degree of deviation from the ideal perovskite structure depends on the radius of the rare earth ion. The cubic symmetry is deformed by the tilting and rotating of the CrO_6_ octahedra.

The magnetic properties of transition metal (TM) ion-doped *X*NbO_3_ on the Nb site are given by the Heisenberg model:

(1) $$H_{m}=-\sum_{ij}xJ_{ij}S_{i}\cdot S_{j}-\sum_{i}D_{i}{\left(S_{i}^{z}\right)}^{2}-g\mu_{B}h\cdot \sum_{i}S_{i},$$

where $S_{i}$ is the Heisenberg spin operator of the TM ion at the site *i*. $J_{ij}$ is the exchange interaction constant between the TM ions, $D_{i}$ is the single-ion anisotropy constant, h is an external magnetic field, *x* is the ion doping concentration.

The magnetization $M=\langle S^{z}\rangle$ is calculated as

(2) $$M=\langle S^{z}\rangle =\frac{1}{N}\sum_{ij}\left[\left(S+0.5\right)\coth\left[\left(S+0.5\right)\beta E_{mij}\right)]-0.5\coth\left(0.5\beta E_{mij}\right)\right],$$

where *S* is the spin value, $\beta =1/k_{B}T$, and $k_{B}$ is the Boltzmann constant. $E_{mij}$ are the spin excitations calculated using the spin Green’s function $G_{ij}=\ll S_{i}^{+};S_{j}^{-}\gg$:

(3) $$E_{ij}=\frac{\langle \left[\left[S_{i}^{+},H_{m}\right],S_{j}^{-}\right]\rangle }{\langle \left[S_{i}^{+},S_{j}^{-}\right]\rangle }.$$

In order to observe the band gap energy we need the s-d model $H_{s-d}=H_{m}+H_{el}+H_{m-el}$. The Hamiltonian of the conduction band electrons $H_{el}$ is given by:

(4) $$H_{el}=\sum_{ij\sigma }t_{ij}c_{i\sigma }^{+}c_{j\sigma },$$

where $t_{ij}$ is the hopping integral, $c_{i\sigma }^{+}$ and $c_{i\sigma }$ are Fermi-creation and -annihilation operators.

The s-d coupling term $H_{m-el}$ reads

(5) $$H_{m-el}=\sum_{i}I_{i}S_{i}s_{i},$$

where *I* is the s-d interaction constant. The spin operators $s_{i}$ of the conduction electrons at site *i* can be expressed as $s_{i}^{+}=c_{i+}^{+}c_{i-}$, $s_{i}^{z}=\left(c_{i+}^{+}c_{i+}-c_{i-}^{+}c_{i-}\right)/2$.

The ferroelectric properties of pure and doped *X*NbO_3_ are described by the transverse Ising model:

(6) $$H_{f}=-\Omega \sum_{i}B_{i}^{x}-\frac{1}{2}\sum_{ij}\left(1-x^{\prime }\right)J_{ij}^{\prime }B_{i}^{z}B_{j}^{z}.$$

The pseudo-spin operator $B_{i}^{z}$ characterizes the two positions of the ferroelectric unit at the lattice point *i*. The exchange pseudo-spin interaction is taken to be ferroelectric, $J_{ij}^{\prime }>0$ for LNO and KNO, and antiferroelectric, $J_{ij}^{\prime }<0$ for ANO and NNO. The dynamics of the model with strength Ω is determined by the operator $B^{x}$. $x^{\prime }$ is the doping concentration by doping on the A site.

The relative polarization *P* is calculated from $\langle B^{z}\rangle$:

(7) $$P=\langle B^{z}\rangle =\frac{1}{2N}\sum_{ij}\tanh\frac{E_{fij}}{2k_{B}T}.$$

$E_{fij}$ is the pseudo-spin excitation energy observed from the poles of the Green’s function ${\tilde{G}}_{ij}=\ll B_{i}^{+};B_{j}^{-}\gg .$

The two subsystems (1) and (6) are coupled through the magnetoelectric coupling *g*:

(8) $$H_{mf}=-g\sum_{iklm}B_{i}^{z}B_{k}^{z}S_{l}\cdot S_{m}.$$

The band gap energy $E_{g}$ of *X*NbO_3_ can be observed from the difference between the valence and conduction bands:

(9) $$E_{g}=\omega^{+}\left(k=0\right)-\omega^{-}\left(k=k_{\sigma }\right)$$

with the electronic energies

(10) $$\omega^{\pm }\left(k\right)=\epsilon_{k}-0.5\sigma I\langle S^{z}\rangle ,$$

which are obtained from the Green’s functions $g_{ij\sigma }=\ll c_{i\sigma };c_{j\sigma }^{+}\gg$, σ=±1. $\epsilon_{k}$ is the conduction band energy in the paramagnetic state, $\langle S^{z}\rangle$—the magnetization, *I*—the s-d interaction constant. 

### 2.2. Numerical Calculations

The numerical results are made using the following model parameters: $J^{Fe-Fe}$ = 510 K, *D* = −2.88 K [37], *I* = 0.2 eV, *I* = 0.2 eV, *g* = 15 K, $J^{\prime }$ = 550 K, Ω = 20 K, S(Fe^3+^) = 5/2, and *S* = 1/2 for the pseudo-spins.

**Figure 2:**
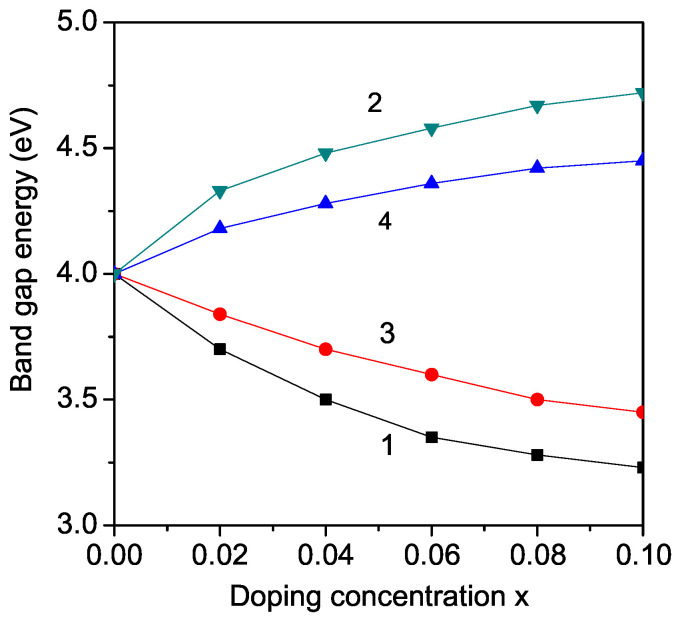
(Color online) Dependence of the band gap energy $E_{g}$ in LiNbO_3_ (LNO) on the doping concentration *x* for different doping ions: (1) Fe at Nb site; (2) Zn at Nb site; (3) Ba at Li site; (4) Fe at Li site. The $E_{g}$ values for *x* = 0.1 are 3.23, 3.45, 4.45, 4.72 eV for curves 1, 3, 4, 2, respectively.

**Figure 3:**
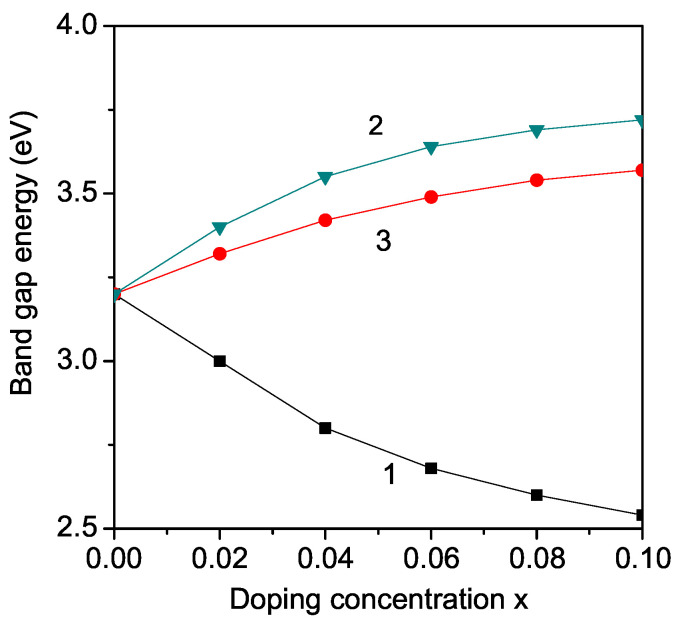
(Color online) Dependence of the band gap energy $E_{g}$ in KNbO_3_ (KNO) on the doping concentration *x* for different doping ions: (1) Fe at Nb site; (2) Zn at Nb site; (3) Ba at K site. The $E_{g}$ values for *x* = 0.1 are 2.54, 3.57, 3.72 eV for curves 1, 3, 2, respectively.

KNO and LNO, which exhibit orthorhombic crystal structure, are not widely used in photocatalytic applications because of their large band gap ($E_{g}$ = 3.2–3.3 eV, and 4 eV, respectively) which is not appropriate for the visible range of the solar spectrum. However, doping of KNO (LNO) may reduce its band gap. The modified band gap can be adjusted in a wide range from 1.1 to 3.8 eV, which represents the ideal visible light absorption band gap (approximately 1.39 eV). Thus, this modified band gap can be used in solar photovoltaics. Therefore, firstly, we calculate, from Equations (9) and (10), the band gap energy $E_{g}$ in transition metal ion-doped KNO (LNO) at the Nb site, for example, Fe, Co, Ni, and Mn. We observe a decrease in the band gap energy $E_{g}$ in both cases KNO and LNO. The results for Fe^3+^ ion doping (*r* = 0.69 $\dot{A}$) at the Nb^5+^ (*r* = 0.78 $\dot{A}$) site are presented in Figure 2 and Figure 3, curves 1. It appears as a compressive strain. The exchange interaction constant between the Fe ions, which is inversely proportional on the lattice parameters, increases with increasing *x*. This leads to enhanced magnetization *M* and reduced band gap $E_{g}$ with increasing dopant (see Equation (10)). Zheng et al. [38], Ait brahim [39] and Maarouf et al. [12] reported a reduced $E_{g}$ in Fe-doped LNO and KNO, too. We also obtain this behavior for other TM ions with ionic radius smaller than that of Nb in qualitative agreement with the experimental data of Maarouf et al. [12] for KNO and of Liang et al. [14], Mamoun et al. [40], and Zainuddin et al. [41] for LNO. The results indicate that the band gap $E_{g}$ is significantly narrowed for all the transition metal-doped LNO and KNO. This decrease is stronger for larger dopant concentration, *x*. The undoped LNO and KNO show absorption only in the UV region of the optical spectrum. For all TM dopants, the absorption is significantly enhanced in the visible region of the optical spectrum. This enhancement in absorption within the visible spectrum is a result of the introduction of TM dopants, which introduce additional energy levels within the band structure of the undoped LNO and KNO materials. These dopants create localized states within the band gap, allowing for the absorption of photons with energies corresponding to the visible range. This broadening of the absorption spectrum significantly improves the materials’ efficiency in harnessing sunlight for energy conversion processes, making them more suitable for applications such as solar cells and photodetectors. Moreover, the tunability of the absorption properties through dopant selection offers further opportunities for optimizing device performance and expanding the functionality of these materials in diverse optoelectronic applications.

By Zn^2+^ ion substitution of the Nb^5+^ ions in KNO or LNO, we observe the opposite behavior, an increase of the band gap energy $E_{g}$ (see Figure 2 and Figure 3, curve 2), in qualitative coincidence with the experimental results of Liang et al. [14] and Zhao et al. [42], respectively. This behavior could be caused by the lattice distortion due to the larger ionic radius of the Zn ion (0.88 $\dot{A}$) in comparison with that of the Nb ion, i.e., there appears to be tensile strain. The exchange interaction constants and magnetization decrease. We obtain an increase in the band gap $E_{g}$ by Mg^2+^ ion (0.86 $\dot{A}$) doping on the Nb site in LNO in qualitative agreement with the experimental data of Huang et al. [25]. Huang et al. [25] also reported differences in the band gap behavior in Fe and Mg-doped LNO, as observed here. Sidorov et al. [20] also reported an increase in $E_{g}$ in Zn and Mg-doped LNO. We can conclude that doping with Zn or Mg ions on the Nb site is not appropriate for application in solar photovoltaics. It must be mentioned, that by substitution of Nb with the same ions in LNO and KNO, the changes in the band gap energy are the same because the relation between the ionic radii of the doping and host (Nb) ions is the same.

There is experimental evidence that the substitution of Li, for example, by Fe in LNO is energetically more preferable than the substitution of Nb by Fe [38]. Therefore, we have calculated the band gap energy $E_{g}$ of Fe-doped LNO in the case that Fe substitutes the Li ion. The ionic radius of the Fe ion is, in this case, larger than that of the Li ion, contrary to the case of the substitution of the Nb ion where the ionic radius of the Fe ion was smaller than that of Nb. The result for the band gap energy in dependence on the Fe doping concentration is presented in Figure 2, curve 4. It can be seen that $E_{g}$ increases with $x^{\prime }$. Mazkad et al. [21] showed that rare earth cations, e.g., Sm in LNO, prefer occupancy of the octahedral sites of Li or Nb.

The more interesting case is the substitution of the K(Li) ions. By Nb ion substitution with TM ions, the ferromagnetic behavior is modified, whereas by substitution of K(Li) it is the semiconducting one. Substitution of the Ba ion into the A-site of the perovskite structure leads to enhanced polarization [30]. Therefore, one can conclude that the Ba ion may help to improve the energy storage performance of these materials. By doping, for example, with the alkaline earth metal Ba^2+^ ion, where the radius (1.49 $\dot{A}$) is smaller compared to that of the K ion (1.52 $\dot{A}$) but larger than that of the Li ion (0.9 $\dot{A}$), different stains appear. In the first case in KNO, we have a compressive strain, in the second one, in LNO, a tensile strain. This leads to different behaviors of the concentration dependence of the band gap $E_{g}$, see Figure 2 and Figure 3, curves 3. In LNO, the band gap $E_{g}$ decreases with increasing Ba dopant, whereas in KNO, $E_{g}$ increases with enhancing Ba ions. This shows the importance of the doping site and the caused strains.

**Figure 4:**
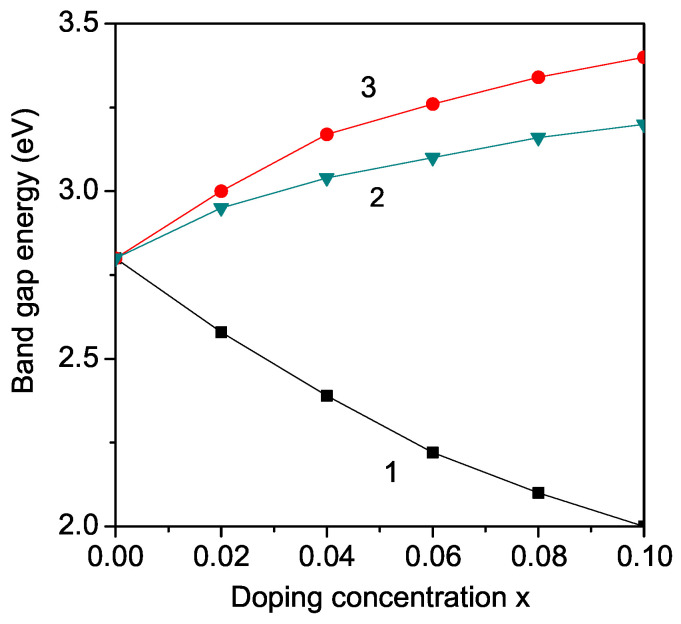
(Color online) Dependence of the band gap energy $E_{g}$ in AgNbO_3_ (ANO) on the doping concentration *x* for different doping ions: (1) Fe at Nb site; (2) Zn at Nb site; (3) Ba at Ag site. The $E_{g}$ values for *x* = 0.1 are 2, 3.2, 3.4 eV for curves 1, 2, 3, respectively.

**Figure 5:**
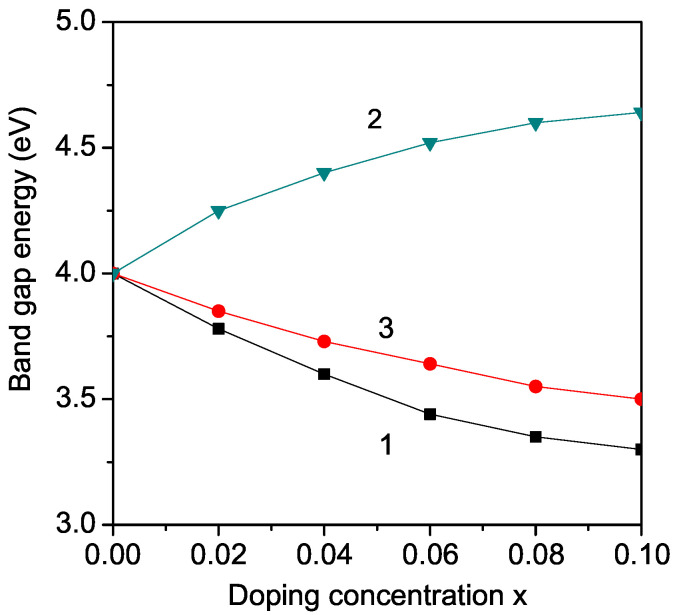
(Color online) Dependence of the band gap energy $E_{g}$ in NaNbO_3_ (NNO) on the doping concentration *x* for different doping ions: (1) Fe at Nb site; (2) Zn at Nb site; (3) Ba at Na site. The $E_{g}$ values for *x* = 0.1 are 3.3, 3.5, 4.64 eV for curves 1, 3, 2, respectively.

Now we will discuss the band gap and polarization tuning of ANO and NNO by different ion doping. They belong to the perovskite family and, at room temperature, exhibit the orthorhombic symmetry with the Pbcm space group [26]. Let us emphasize, that because these compounds are antiferroelectric, we have to use a negative exchange pseudo-spin interaction constant $J^{\prime }$ in Equation (6). The doping of ANO and NNO with Fe, Ni, Mn, Co or Zn, Mg on the Nb site leads to the same results as by LNO or KNO, because the radius relation between the doping and host ions remains unchanged (see Figure 4 and Figure 5). This is confirmed by experimental data, for example, $E_{g}$ increases in NNO with Mg and Ca doping [32], or decreases with Co and La [24], or Mn and Ni [43]. By ion substitution on the Ag or Na site, again differences appear between ANO and NNO (see Figure 3 and Figure 4) as well as between them and LNO or KNO (see Figure 2, Figure 3, Figure 4 and Figure 5). Unfortunately, there is no experimental data for the tuning of $E_{g}$ in Ba-doped *X*NbO_3_ compounds. It should be mentioned that the experimental data of Han et al. [30] suggested the enhanced electric properties of ANO after Ba modification, making it a promising candidate for energy storage applications.

**Figure 6:**
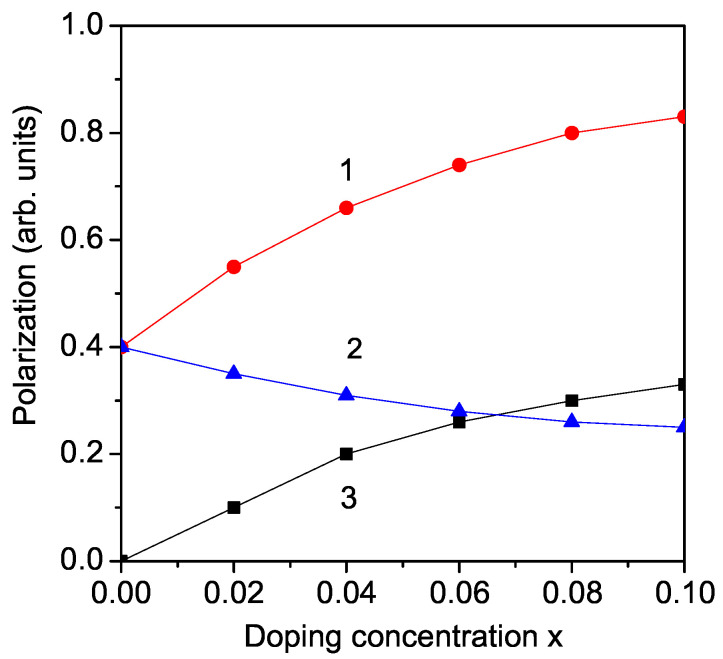
(Color online) Dependence of the polarization *P* on the doping concentration *x* for different doping ions: (1) Ba at K site in KNbO_3_ (KNO); (2) Fe at Nb site in KNO; (3) Ba at Na site in NaNbO_3_ (NNO).

Modifying the A and B site cations, one can change the electrical characteristics useful for a variety of applications, such as photovoltaic ones and energy storage capabilities of these materials [34]. Zhang et al. [11] have investigated the optical band gap $E_{g}$ and the ferroelectricity in La/Co co-doped KNO, K_1−*x*_La_*x*_Nb_1−*x*_Co_*x*_O_3_. The results reveal that La doping improves the ferroelectric properties whereas Co doping significantly reduces the $E_{g}$. Enhanced antiferroelectric phase stability in La-doped ANO is reported by Gao et al. [33]. A high ferroelectric polarization (due to the Ba ion doping) with a narrow optical band gap (due to the Ni doping ions) is observed in Ba/Ni co-doped KNO (K_1−*x*_Ba_*x*_)(Nb_1−*x*_/2Ni_*x*_/2)O_3_, by Song et al. [44]. In Figure 6, we have demonstrated the Ba ion doping dependence of the polarization *P* (calculated from Equation (7)) in KNO (curve 1) and NNO (curve 2). It can be seen that *P* increases with the Ba dopant. Curve 3 shows the reduced ferroelectricity by Fe doping in KNO on the Nb site. KNO is ferroelectric, and through the Fe doping, there appears to be a weak ferromagnetism, and so the compound is now multiferroic. With increasing Fe dopant, the ferromagnetism increases, whereas the polarization decreases due to the magnetoelectric coupling and the increasing intrinsic oxygen vacancies. This is in qualitative coincidence with the experimental data [11,45].

## 3. Conclusions

In conclusion, it is observed that the behavior of the band gap energy $E_{g}$ and the polarization *P* of LNO, KNO, ANO, and NNO compounds depends on the nature of the dopant. Both properties are studied by doping with different ions at different A or/and B sites for *x* = 0–0.1. It is shown that for all substances, $E_{g}$ decreases or increases with increasing Fe or Zn doping concentration (at the Nb site), respectively. But the substitution, for example, of Ba at the A site leads to different behaviors of these materials. The polarization *P* increases by Ba doping at the A site and decreases by Fe substitution at the Nb site. It is discussed that in order to be appropriate for photovoltaic and energy storage applications, the compounds should have a large polarization and reduced band gap in comparison with the undoped ones. The results suggest that the electric properties of *X*NbO_3_ can be largely tuned after Ba modification, making it a promising candidate for energy storage applications. Moreover, the reduced band gap energy observed by doping with transition metal or Ba ions could be used for photovoltaic applications. By Ba/Fe and Ba/Ni co-doping (Ba at the A site, Fe, Ni at the B site) we observe both an enhanced polarization and a reduced band gap.

## Data Availability

Derived data supporting the findings of this study are available from the corresponding author upon reasonable request.
